# Deciphering pathological behavior of pediatric medullary thyroid cancer from single-cell perspective

**DOI:** 10.7717/peerj.15546

**Published:** 2023-09-20

**Authors:** De-qian Chen, En-qing Zhou, Hui-fen Chen, Yong Zhan, Chun-Jing Ye, Yi Li, Shu-yang Dai, Jun-feng Wang, Lian Chen, Kui-ran Dong, Rui Dong

**Affiliations:** 1 Department of Pediatric Surgery, Children’s Hospital of Fudan University, and Shanghai Key Laboratory of Birth Defect, Fudan University, Shanghai, China; 2 Department of Pathology, Children’s Hospital of Fudan University, Fudan University, Shanghai, China

**Keywords:** Medullary thyroid cancer, Sporadic medullary thyroid cancer, Pediatric tumor microenvironment, Single-cell transciptome sequencing, Whole-exome analysis

## Abstract

**Background:**

Pediatric medullary thyroid cancer (MTC) is one of the rare pediatric endocrine neoplasms. Derived from C cells of thyroid glands, MTC is more aggressive and more prompt to metastasis than other types of pediatric thyroid cancer. The mechanism remains unclear.

**Methods:**

We performed single-cell transcriptome sequencing on the samples of the primary tumor and metastases lymph nodes from one patient diagnosed with MTC, and it is the first single-cell transcriptome sequencing data of pediatric MTC. In addition, whole exome sequencing was performed and peripheral blood was regarded as a normal reference. All cells that passed quality control were merged and analyzed in R to discover the association between tumor cells and their microenvironment as well as tumor pathogenesis.

**Results:**

We first described the landscape of the single-cell atlas of MTC and studied the interaction between the tumor cell and its microenvironment. C cells, identified as tumor cells, and T cells, as the dominant participant in the tumor microenvironment, were particularly discussed in their development and interactions. In addition, the WES signature of tumor cells and their microenvironment were also described. Actively immune interactions were found, indicating B cells, T cells and myeloid cells were all actively participating in immune reaction in MTC. T cells, as the major components of the tumor microenvironment, proliferated in MTC and could be divided into clusters that expressed proliferation, immune effectiveness, and naive markers separately.

## Introduction

Pediatric thyroid cancer is the most common endocrine tumor in children (Pereira et al., 2020). Papillary thyroid cancer (PTC) is the most frequent subtype accounting for most of cases, while other types including MTC make up only 5% of cases (Ohira et al., 2018; Castinetti & Taïeb, 2021). In pediatric MTC patients, the majority are male, and the average age of diagnosis is younger than 4 years old (Dermody, Walls & Harley, 2016). It is generally acknowledged that the tumor cells of MTC are derived from thyroid C cells, which are of neuroendocrine origin and have functions in both the nervous and endocrine systems.

Compared to other subtypes of pediatric thyroid cancer (De Carlos Artajo et al., 2021), MTC is considered more malignant and prone to metastasis (Wells Jr et al., 2015; Wu, Li & Zheng, 2022; Roman, Lin & Sosa, 2006). Previous studies have reported clinical traits of MTC among adult patients. Sporadic variants account for about 75% of all adult MTC, and mutations were identified not only in the RET gene, in which single and multiple point mutations as well as small deletions/deletions–insertions are described, but also other cancer-related genes, the alteration of which has been reported either alone or in association with the RET/RAS drivers (Minna et al., 2022). Although the majority of pediatric MTC cases are associated with multiple endocrine neoplasia (MEN) or a known familial history of MTC (Kotwal et al., 2021), the mechanism underlying its aggressive behavior remains unclear.

Single-cell sequencing technology has recently emerged as a powerful tool for depicting transcriptome patterns of tumors at the single-cell resolution. This technology enables further exploration of inter-tumor heterogeneity by comparing bulk tissue transcriptome sequencing (Dong et al., 2020; Luo et al., 2021), and provides us a valuable opportunity to explore the underlying pathogenesis and metastasis mechanism of MTC. Therefore, we aim to analyze single-cell sequencing data of primary and metastatic lesions of MTC patients and to combine WES data for a comprehensive understanding of the disease.

Through the single-cell atlas, we can gain a better understanding of the relationship between the incidence of pediatric MTC and its pedigree mutations, tumor characteristics, ecosystem, and the possible genes affecting tumor development. Such insights could provide us potential inspirations for future treatment strategies.

## Materials & Methods

### Human tumor specimens

For single-cell mapping, a patient with MTC was enrolled at the Children’s Hospital of Fudan University. We performed bulk whole-exome sequencing using tumor tissues and paired PBMC samples. In addition, whole genome sequencing was performed using PBMC samples of this patient and his parents and siblings. This study was reviewed and approved by the Children’s Hospital of Fudan University Institutional Review Board (protocol no. 2020 (422)). The written informed consent was obtained from each patient or the guardians of the participant who was <18 years old.

### Bulk whole exome Sequencing and analysis

Total DNA from surgical resections and matched peripheral blood mononuclear cells (PBMC) was extracted using AllPrep DNA/RNA Mini Kit (Qiagen, Hilden, Germany) and Universal Genomic DNA Kit (CWBIO,China) following the manufacturer’s instructions, respectively. Degradation and contamination of DNA were tested with 1% agarose gels, while the concentration and quality of DNA were measured by Qubit dsDNA HS Assay Kit (Thermo Fisher Scientific, Waltham, MA, USA). Qualified tumor samples and matched PBMC were used for the construction of the sequencing library. Next, enzymatic gDNA libraries were prepared using Twist Target Enrichment Kits (Twist, USA) by following the manufacturer’s instructions. WES libraries, generated according to Twist Fast Hybridization Target Enrichment Protocol (Twist, South San Francisco, CA, USA) were then run on NovaSeq6000 (Illumina, USA) to achieve a minimum of 150× on target coverage for the library of per sample. The raw output of Illumina sequencing data was processed and converted to FASTQ format for subsequent analysis. Variants were called using the Genome Analysis Toolkit (GATK) and annotated with ANNOVAR.

### Single-cell dissociation

Biopsies were kept in MACS Tissue Storage Solution (Miltenyi Biotec) until processing. Briefly, samples were first washed with phosphate-buffered saline (PBS), minced into small pieces (one mm^3^) on ice. Then samples were digested in 300 U/mL collagenase II (Worthington), 100 U/mL collagenase IV (Worthington) and 10% FBS in DMEM at 37 °C with agitation for 30 min. After digestion, samples were filtered through a 70 µm cell strainer and centrifuged at 400g for 5 min. After removing the supernatant, the pelleted cells were suspended in erythrocyte lysis buffer (Miltenyi Biotec) to eliminate erythrocytes and washed with PBS with 0.04% BSA. The cells pellets were then re-filtered through a 35 µm cell strainer. Then, the dissociated cells were stained with AO and PI to measure viability by Countstar Fluorescence Cell Analyzer.

### Pre-processing of single cell RNA-seq data

Raw sequencing data were converted to FASTQ files with Illumina bcl2fastq, version 2.19.1, and data were aligned to the human genome reference sequence (GRCH38). The CellRanger (10X Genomics, 3.1.0 version) analysis pipeline was used for sample demultiplexing, barcode processing, and single-cell 3′ gene counting to generate a digital gene-cell matrix from these data. The gene expression matrix was then processed and analyzed by Seurat (version 4.0.2) and an R toolkit (https://github.com/satijalab/seurat), using the software R (version 4.2.1; R Core Team, 2022). We performed Seurat-based filtering of cells based on the number of detected genes per cell (250–6,000) and the percentage of mitochondrial genes expressed <7.5%. The doublets algorithm was then applied to distinguish the potential doublet cells. The doublet score for every single cell was calculated, and the threshold of expected doublet_rate was defaulted as 0.02 in calculating the bimodal distribution. Next, we removed all mitochondrial genes from the matrix. Following quality control, 12,830 high-quality cells were retained with an average of 1,418 genes detected per cell. The statistical power of this experimental design, calculated in RNASeqPower (https://rodrigo-arcoverde.shinyapps.io/rnaseq_power_calc/) is 1. Variations in the calculation of power analysis were below: the sequencing depth was 150; coefficient of variation was 0.4; alpha was 0.05; effect was 2; each cell was regarded as one sample in single-cell RNA sequencing and the sample size would be 9,113, which was same as cell numbers in scRNA data.

### Single cell RNA-seq analysis and statistic

Clustering analysis was performed to define major cell types, following evaluation of the expression level of typical cell type markers. We defined major cell types based on well-known cell markers: fibroblasts (*TAGLN, COL1A1, SPARC, ACTA2, MFAP5*), endothelial cells (*ENG, CLDN5, PECAM1, ICAM2, CD34*), B cells (*CD79A, CD79B, CD19*), myeloid cells (*CD14, CD68, AIF1, CSF1R, TYROBP*), T cells (*CD2, CD3D, CD3E, CD3G*), epithelial cells (*EPCAM, KRT8, KRT18*), C cells (*CALCA*). Next, we performed both inferCNV analysis and copyKAT algorithm to verify our definition of C cells recognized as malignant tumor cells. To explore tumor cell lineages or states, we applied NMF to extract the transcriptional programs of malignant cells of the primary tumors and their metastasis counterparts. Then, we performed monocle algorithm to infer the origin of tumor cells and differentiation as well as the proliferation of T cell.

## Results

### Landscape of pediatric MTC

**Figure 1:**
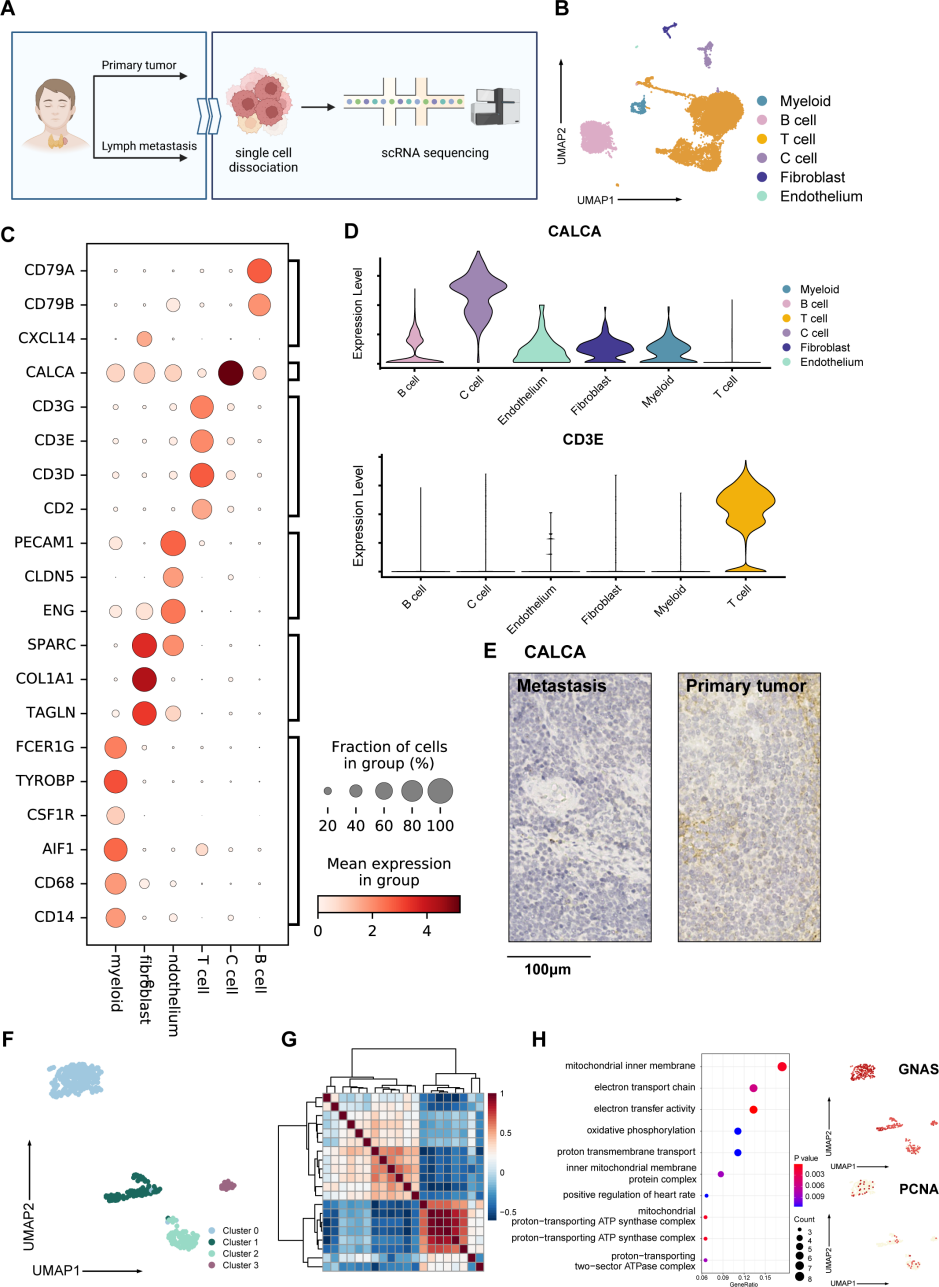
Overall landscape of MTC tumor. (A) Process of samples from a patient diagnosed with madullary thyroid cancer tumors; samples from surgical excision were conducted with single-cell sequencing, hematoxylin-eosin staining, and immunohistochemistry staining. (B) Umap plot of single-cell atlas of 12,830 cells from the primary tumor and lymph node metastases. (C) Dotplot shows the expression of well-known markers in tumor samples. (D) Upper panel: Vlnplot exhibits the expression of CALCA, which is specifically expressed within C cells; lower panel: Vlnplot exhibits the expression of *CD3E*, which is specifically expressed within T cells. (E) Immunohistochemistry staining of *CALCA* results of the primary tumor (left) and metastasis lymph nodes (right) validated pathological diagnosis of medullary thyroid cancer, scale bar: 100 µm. (F) Umap plot shows 427 C cells and re-clusters as 4 clusters. (G) Heatmap exhibits that C cells could be divided into 3 meta-programs according to the correlation between NMF modules. (H) Right panel: Dotplot shows enriched pathways from the result of GO analysis of genes expressed in cells from meta program A. Left panel: Umap plots show specifically expressed *GNAS* and *PCNA* in C cells. Created with BioRender.com.

With informed consent obtained, we collected a pair of samples from an 11-year-old male diagnosed with sporadic MTC at Children’s Hospital of Fudan University. The samples included the primary tumor and lymph node metastases from surgical resection, which were then conducted single-cell sequencing. The patient’s MTC was staged as T1N1bM1 according to the AJCC 8th edition criteria (Perrier, Brierley & Tuttle, 2018) (Fig. 1A). After surgery, tumor samples and peripheral blood samples were also collected for WES (Fig. 1A) to identify potential germline and somatic mutations.

Following quality control, we obtained 12,830 high-quality cells from primary tumor and lymph node metastasis, with an average Ncounts_RNA of 3,398 per cell and an average of 1,418 features per cell. (Fig. S1A). We identified 16 clusters after merging and removing the batch effect between samples (Korsunsky et al., 2019), which we defined as C cells, T cells, B cells, fibroblasts, endothelial cells, and myeloid cells (Fig. 1B). The cell proportion between primary tumor and lymph metastasis did not significantly change (Fig. S1B). Using well-known cell markers, we identified different cell types (Fig. 1C) and validated our definition by scoring all cells with gene sets (Fig. S1C). We defined C cells as malignant cells and found that there were no significant copy number variation (CNV) differences between C cells and other cell types (Fig. S1D). To validate our definition of malignant cells, we used the copyKAT algorithm (Gao et al., 2021) and found that most C cells were recognized as aneuploids (Figd. S1E and S1F).

The expression of *CALCA*, a classical marker of neuroendocrine cells, was significant in our defined C cells (Fig. 1D). This expression pattern matched the neuroendocrine origin reported in previous studies (Wang et al., 2018), and was verified by immunohistochemistry results (Fig. 1E), indicating that the single-cell data could basically reflect the pathological characteristics of the tumor. A total of 427 C cells from both primary tumor and lymph node metastasis samples. After conducted normalization and re-clustering, the C cells could be identified as four clusters (Fig. 1F). Differential gene expression analysis and dimensional reduction analysis, such as non-negative matrix factorization (NMF), were subsequently performed on these clusters (Fig. 1G, Table S1). The gene ontology (GO) analysis of differentially expressed genes (DEGs, Fig. 1H) revealed that cluster 0 cells, which expressed genes such as *MIF* and *GNAS*, were associated with the electron transport chain, oxidative phosphorylation, and ATP synthesis. DEGs of cluster 1 were characterized by *CLDN7* and *LGALS3BP*.

GO analysis also revealed an active interaction between malignant cells and the immune component of tumor microenvironment (TME). Cluster 2 cells expressed classic T cell markers such as *TIGIT* and *ITGB1*, as well as specific neuroendocrine marker *CALCA* (Camacho et al., 2013). Cluster 3 was characterized by the expression of *PCNA, RFC2, SLBP,* and *MRPL36*, indicating a higher proportion of proliferating cells compared to normal C cells, which typically exhibit a lower level of proliferation (Bradford & Jin, 2021; Piao et al., 2009; Arias & Walter, 2006; Hess et al., 2011) (Fig. S1G). Trajectory analysis (Cao et al., 2019) also suggested that metabolically active cluster 0 might have two differentiation directions, one involving interaction with immune cells and the other involving an active proliferation state (Fig. S1H).

### Tumor genesis and microenvironment interactions

**Figure 2:**
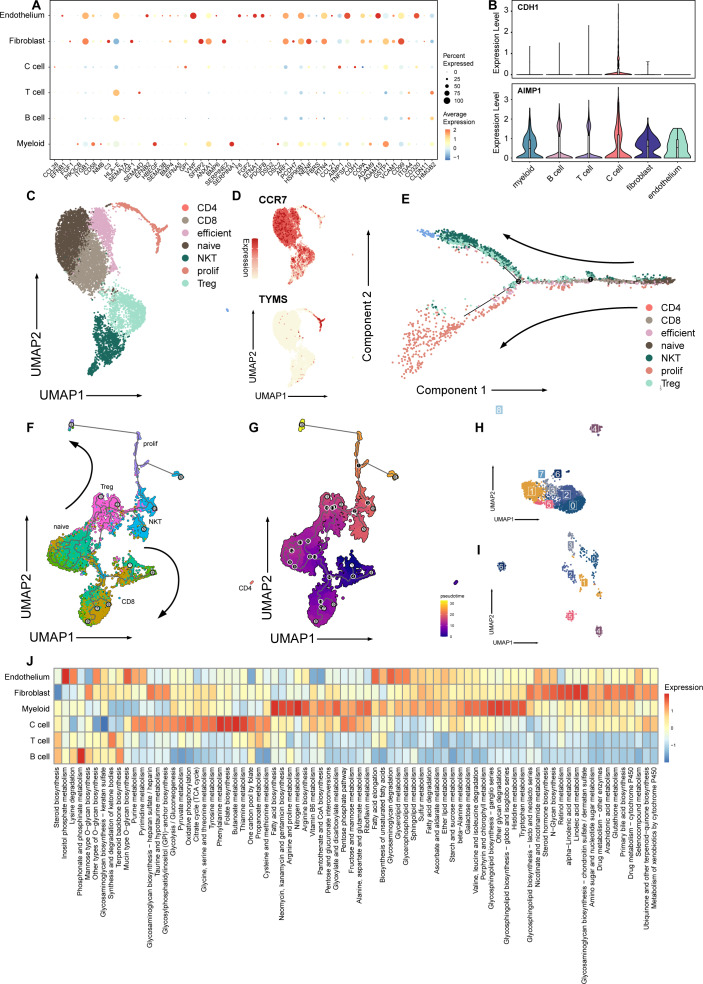
Signature and relationship between tumor cells and T cells. (A) Dotplot shows the expression of genes associated with cell cycle in all cell types. (B) Vlnplots show expression patterns of *CDH1* and *AIMP1* among cell types. (C) Umap plot of seven subtypes in T cells. (D) Umap plot of expression of *CCR7* and *TYMS* In T cells. (E) Trajectory analysis result exhibits two possible differential traces in T cells, the direction of two differential paths is indicated by black arrows. (F) Umap plot shows the pseudotime trajectory of seven T cell subtypes, and the direction of two differential paths was indicated by black arrows. (G) Umap plot shows pseudotime calculation among T cells. (H) Umap plot shows B cells and re-clusters as eight clusters. (I) Umap plot shows myeloid cells and re-clusters as seven clusters. (J) Heatmap plot shows metabolism pathways enriched in different celltypes of medullary thyroid cancer.

The Nichenet algorithm (Browaeys, Saelens & Saeys, 2020) was conducted to explore ligand–receptor interactions of cell cycle-related genes within all cell types (Table S2), within the aim of obtaining a further understanding of the transcription factors associated with tumor pathogenesis and proliferation (Fig. 2A). The result indicated that receptors expressed in C cells were characterized by the upregulated expression of *AIMP1, CDH1, GMFB*, and *GPI* (Figs. 2B–2C).

A previous study has reported that MTC is an immunologically active tumor  (Pozdeyev et al., 2020). To explore the interaction between tumor and immune TME, we extracted T cells, B cells and myeloid cells for further study.

Next, we explored the influence of the immune TME. A total of 9,113 T cells were identified and re-clustered into eight clusters. These cells were classified into seven subtypes based on their proliferation and differentiation status (Fig. 2C), including CD4+, CD8+, efficient T cells, naive T cells, natural killing T cells (NKTs), proliferating T cells, and regulatory T cells (Tregs) (Guo et al., 2018; Zhang et al., 2021; Zheng et al., 2021). Among these T cells, markers of proliferating cells and naïve T cells were widely expressed with relatively high intensity (Fig. 2D). In terms of proliferation score and average gene expression among T cells in different subtype, a similar expression pattern was observed, where the proliferating subtype of T cells exhibited the highest proliferation score and the highest number of gene expression as well (Fig. S2A). Similarly, after NMF analysis, T cells were mainly divided into three modules (Fig. S2B, Table S3). GO analysis showed that these meta-modules were related to T cell effects, cell proliferation, and T cell differentiation, respectively (Fig. S2C). The results of trajectory analysis also showed that T cells could differentiate into proliferation active status, effector, and exhibited different stages and directions during differentiation process from naïve T cells to root states (Fig. 2E, Fig. S2D). The conclusion was validified using the monocle3 algorithm (Fig. 2F, Fig. 2G).

When analyzing other immune component of microenvironment, we have identified 2,763 B cells and 340 myeloid cells. B cells were re-clustered and divided into nine clusters (Fig. 2H, Fig. 2I). Two vague differentiation directions were observed, characterized by expression of *CD79A* and *CD79B* (Fig. S2F). In contrast to T cells, B cells exhibited no significant difference in cycling proportions between primary tumor and metastasis (Fig. S2E).

In addition, we utilized scMetabolism to investigate the metabolic pathways among different cell types and observed specific enrichment patterns. As result demonstrated, pathways associated with the Warburg effect were enriched in malignant cells, whereas pathways related to lipid metabolism were identified in myeloid cells (Liberti & Locasale, 2016) (Fig. 2J).

### WES signature of MTC

In the context of MTC cases being predominantly associated with familial inheritance, it becomes necessary to distinguish between sporadic and hereditary MTC. Therefore, WES was conducted on primary MTC tumor, lymph metastasis and peripheral blood samples in order to identify possible somatic and germline mutations.

**Figure 3:**
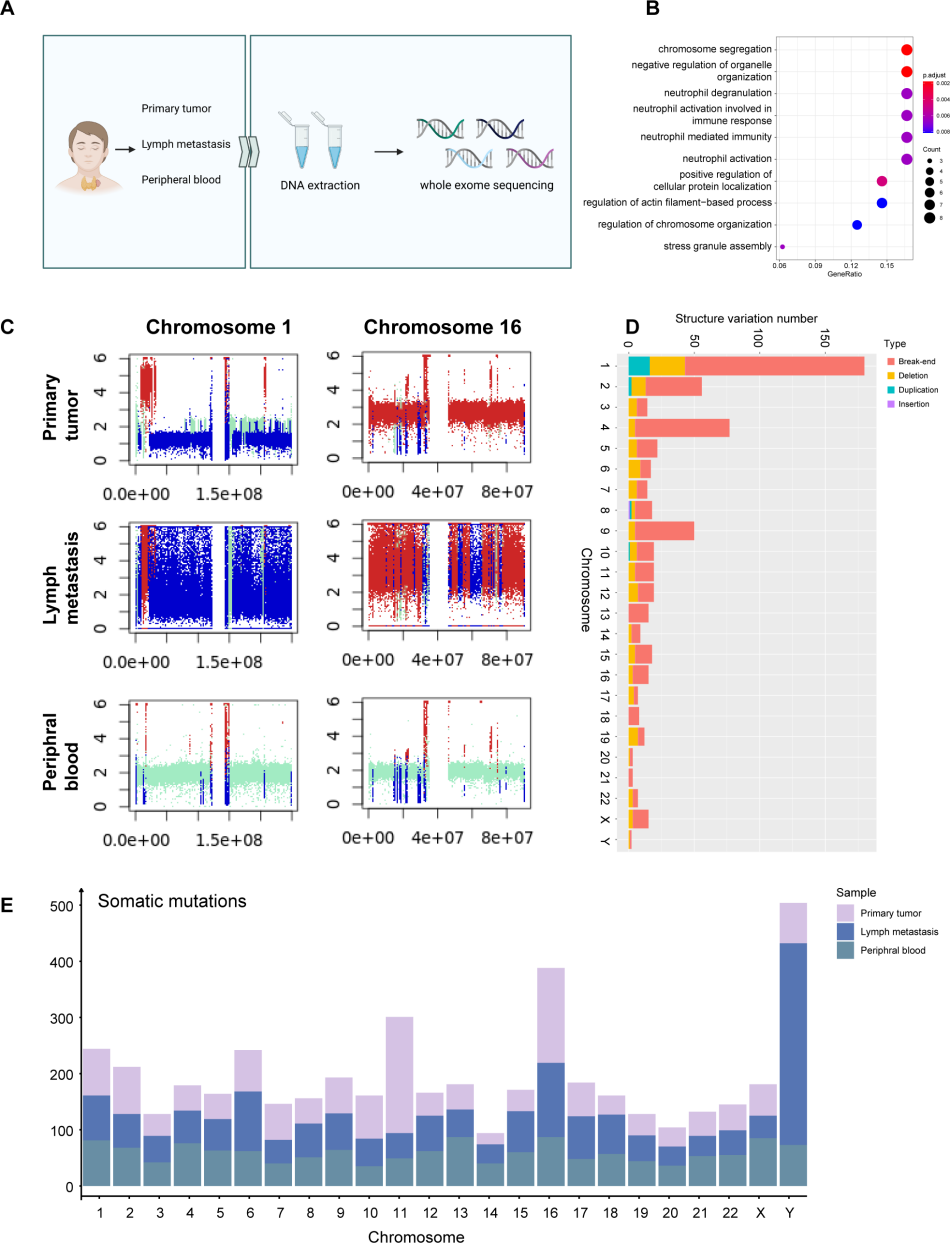
WES signature of MTC. (A) Process of WES applying on primary tumor sample, metastases lymph nodes, and peripheral blood samples. (B) Dotplot shows the top 10 most enriched pathways of GO analysis on somatic mutations of tumor samples. (C) Plots of chromosome 1 and chromosome 16 of the primary tumor sample, metastases lymph nodes, and para-tumor sample showing copy number variation status indicate that deletion occurred massively in chromosome 1 while duplication mostly in chromosome 16. Different colors are used to indicate alteration types; red represents gain, blue represents loss, and cran represents neutral. The *x*-axis shows the predicted copy number, and the *y*-axis indicates the position based on the number of base pairs in the chromosomes. (D) Barplot shows structure variation numbers obtained on each chromosome in the primary tumor sample, and different types are distinguished by colors. Somatic variation numbers of chromosomes 1 and 16 are quoted with frame. (E) Barplot shows the number of somatic mutations on each chromosome in each sample, samples are distinguished by colors.

WES of 100x depth was conducted on primary tumor and metastatic lymph nodes, with samples from the peripheral blood being regarded as normal control (Fig. 3B). Somatic mutation analysis revealed a total of 717 deletion mutations, 1,479 insertion mutations, and 326 single nucleotide polymorphisms (SNPs, Fig. S3A). Among these mutations, there were no identified mutations on exons of RET or ERK gene. The number of somatic mutations in the metastatic sample increased significantly when compared to primary tumors. Notably, *TPR* and *EIF3A* were among the mutated genes, and previous studies have reported that these genes being associated with tumorigenesis and progression. Furthermore, the presence of mutations in *ACTB* in both tumor and metastatic samples suggested an association with the impaired ability of tumor cells to repair DNA damage (Fig. 3B, Fig. S3B).

Comparing CNV patterns among chromosomes, it was observed that chromosomes 1, 9 and 15 had more loss mutations of CNV, while chromosomes 2, and 16 had higher gain mutations than other chromosomes (Fig. S3C). Structure variations (SV) exhibited partially different patterns, while chromosomes 1 and 16 exhibiting a higher incidence of SV (Fig. 3C). While chromosomes 2, 9, and 15 exhibited more CNV mutations (Fig. S3D), there was no significant difference in the proportion of SV (Fig. S3C) associated with genes located on chromosomes 1, and 16.

In the SV analysis of tumors, mild duplication and deletion were identified in chromosomes 5 and 14, which correspond to WES results (Fig. 3D). A total of 1,592 germline mutations were observed and the number of somatic mutations was 3,073. The number of somatic mutations was significantly higher in metastasis than in primary tumors and peripheral blood, consistent with the clinical features of higher malignancy in metastatic cells (Fig. 3E).

## Discussion

According to existing studies, MTC was a rare malignancy among pediatric patients (Francis et al., 2015), with most cases being hereditary and associated with MEN or known family history of MTC (Dermody, Walls & Harley, 2016). However, the landscape of sporadic pediatric MTC remains unclear. To determine the diagnosis of sporadic MTC, WES was conducted to exclude any possible relation with heredity factors. As expected, the WES results indicated no somatic or germline mutations in patient’s tumor sample and peripheral blood, and we observed the absence of pheochromocytoma and related family history of MTC, confirming the diagnosis of pediatric sporadic MTC.

Both pediatric tumors and MTC has been reported with fewer somatic mutations (Gao et al., 2021; Agrawal et al., 2013), which lead to a vague CNV landscape. Therefore, the copyKAT algorithm was conducted as a complementary method to verify the definition of malignant cells. Interestingly, the algorithm also identified some T cells as aneuploids, suggesting an active T cell reaction between the tumor and its TME (Pozdeyev et al., 2020).

Significant inter-tumor heterogeneity was found within tumor cells. GO analysis of DEGs revealed that cluster 0 exhibited an upregulated metabolic state that could promote tumor growth. DEGs upregulated in cluster 1 cells, such as *CLDN7* and *LGALS3BP*, exhibited a more generous expression pattern among different types of malignancies (Hou et al., 2020; Wu et al., 2018). The genes listed above might be related to the cytotoxicity of NK cells and lymphokine-activated killer cells (Song et al., 2021). Being identified as a mixture of classical T cell and C cell markers, we have eliminated any possible doublets before downstream analysis, suggesting that cells in cluster 2 may be actively interacting with T cells, which has been reported by previous studies (Pozdeyev et al., 2020). As discussed above, DEGs among clusters and correspondent GO results indicated the existence of inter-tumoral heterogeneity, which could promote tumor proliferation and metastasis. The relatively high proportion of T cells also suggested the potential interaction between tumor cells and the immune components of TME (Garcia-Alvarez et al., 2022).

Among genes obtained from the Nichenet algorithm that specifically upregulated in C cells, *CDH1* would act as a tumor suppressor gene encoding a classical cadherin. Its inactivation is often associated with the promotion of tumor proliferation, invasion, and metastases (Gamble, Heller & Davis, 2021). Additionally, *GMFB*, the other marker that has been identified in C cells by Nichenet, may participate in pathways including actin binding and frosty factor activity (Li et al., 2010).

When exploring interaction between tumor and its microenvironment components, the proportion of T cells subtypes significantly varied among samples, with a higher proportion of NKT and regulation T cells in primary tumor sample than in metastasis sample. This would indicate an active cell-mediated immune reaction in tumor microenvironment (Terabe & Berzofsky, 2018; Mair et al., 2022), which was suggested by GO result and interactions from Nichenet algorithm as well.

Although B cells and myeloid cells occupied a relatively small portion of TME compositions, previous studies have reported their significance in tumor development, progression, and metastasis. Among all B cells, cluster 6 was identified as naïve status, without any specific differentiation destinations. Previous studies have reported that B cells forming tertiary lymphoid structures could play an important role in tumor immune (Fridman et al., 2022). A small proportion of B cells with similar features was identified in primary MTC data, indicating that this structure may also exist in primary MTC and played a significant role in immune activities in MTC. These results suggested that T cells were actively activated and proliferated, indicating their function in impeding tumor progress. However, it was still unclear what specific role other immune cells in TME, including B cells and myeloid cells, played during tumor progression in MTC.

Indeed, metabolic pathways enriched by scMetabolism suggested an activated metabolism of tumor cells (Liberti & Locasale, 2016) as well as functional myeloid cells (Dou & Fang, 2021). However, no significant difference in the enrichment of metabolism pathways was observed between primary and metastasis.

WES analysis was conducted on primary tumor and metastasis sample immediately after surgery to determine whether the MTC was sporadic or familial subtype. Additionally, the WES data was expecting to identify more somatic or germline mutations than single-cell data, which could provide valuable insights into the interaction between impact factors and tumor progression. Compared to primary tumors, the number of somatic mutations identified in the metastatic sample was significantly higher. Previous studies have reported that *TPR* and *EIF3A* would be associated with tumorigenesis and progression  (Kosar et al., 2021; Yin et al., 2018). This suggested that metastatic tumor cells might accumulate more mutations than their primary counterparts, which enhanced their ability of proliferation and aggression (Accardo et al., 2017). Mutations in *ACTB* were identified in both primary tumors and metastasis, suggesting that they could be associated with impaired DNA damage repair mechanisms (Mercatelli et al., 2021).

The WES analysis revealed that there were CNV and SV mutations present in tumor cells. The differences in the pattern of SVs and somatic mutations, which are characterized by a higher incidence of mutations and a lower incidence of SVs in sex chromosomes, suggest heterogeneity in SVs and somatic mutations. Furthermore, it indicates that SVs may contribute less to the progression of MTC than somatic mutations. Simultaneously, there still existed a considerable proportion of germline mutations that require further investigation.

## Conclusions

This study provided an overview of the landscape of pediatric sporadic MTC, analyzed the TME of pediatric MTC, and identified the pathological behavior of pediatric MTC at the single-cell perspective. Tumor cells were observed to differentiate into two terminals that related to tumor metastasis and tumor proliferation, separately. Furthermore, we discovered active interactions between the TME and tumor cells, and identified several differentiation trajectories of immune components, indicating their significant roles in tumor progression. These findings improved our understanding of pediatric MTC and its pathological behavior.

However, a major limitation of this study was the difficulty in obtaining surgical samples due to the low incidence of pediatric MTC. In future studies, we aim to obtain more MTC samples and succeed in MTC cell culture to validate the findings in the single-cell atlas and investigate further the molecular mechanism underlying pediatric MTC.

##  Supplemental Information

10.7717/peerj.15546/supp-1 Figure S1 Quality control and validation of celltype definition (A) Vlnplots show results of quality control. Upper panel: Vlnplot shows UMI numbers of 3,398 per cell; Downstream panel: Vlnplot shows an average number of 1,418 genes detected in a single cell. (B) Barplot shows proportion of different cell types in primary tumor and metastasis lymph nodes. (C) Upper panel: Umap plots exhibit results of scoring using gene sets of well-known cell type specific markers in primary tumor sample. Downstream panel: Umap plots exhibit results of scoring using gene sets of well-known cell type specific markers in metastasis tumor sample. (D) Left panel: inferCNV result of primary tumor; Right panel: inferCNV result of metastasis lymph tumor. (E) Left panel: Umap plot shows copyKAT prediction result of primary tumor and umap plot shows identification of cell types in primary tumor. Right panel: Umap plot shows copyKAT prediction result of metastasis lymph node and umap plot shows identification of cell types in metastasis lymph node. (F) Heatmap plot shows cNMF results. (G) Dotplot of differential expressed genes in clusters of C cells. (H) Trajectory analysis of C cells of which differentiation directions are indicated by black arrows.

10.7717/peerj.15546/supp-2 Figure S2 Differentiation directions of tumor and its microenvironment (A) Vlnplots show proliferation state and average gene number of T cell subtypes. (B) Heatmap exhibits that T cells could be divided into 3 meta-programs according to correlation between NMF modules. (C) Barplots show GO result of 3 meta-programs from NMF. Upper panel: module A; middle panel: module B; lower panel: module C. (D) Trajectory analysis results of T cells indicate that subtypes, distinguished by colors, locate in different position of differentiation stages. (E) Barplot exhibits proportion of cells in different phase of cell cycle from primary tumor and metastasis. Proportion of T cells, B cells and myeloid cells are shown separately. (F)Umap plots show expression of *JCHAIN*, *IGLL1*, *CD79B* and *CD79A* in B cells, showing an obscure tendency in B cell differentiation. (G) Umap shows pseudotime result of B cells. (H) Umap shows pseudotime result of myeloid cells.

10.7717/peerj.15546/supp-3 Figure S3 WES somatic mutation patterns related to SV (A) Oncoplot shows mostly mutated 20 genes detected among primary tumor, lymph metastases, and para-tumor samples. (B) Vlnplots of expression of ACTB, EIF3A, and TPR in single-cell data, comparing primary tumor sample with metastasis lymph nodes. (C) Plots of chromosome 2, chromosome 9, and chromosome 15 of the primary tumor sample, metastases lymph nodes, and para-tumor sample show CNV status. (D) Barplots describe SV numbers of each chromosome in metastases lymph node and para tumor samples, and SV types are distinguished by colors.

10.7717/peerj.15546/supp-4 Table S1 Top 50 genes of 3 meta-module in C cells after NMF analysis

10.7717/peerj.15546/supp-5 Table S2 Ligand_receptor network that identified being up-regulated in Nichenet

10.7717/peerj.15546/supp-6 Table S3 Top 50 genes of 3 meta-module in T cells after NMF analysis

## Additional Information and Declarations

### Data Availability

The following information was supplied regarding data availability: The data is available at the GSA for Human: HRA003575.
https://ngdc.cncb.ac.cn/gsa-human/browse/HRA003575.
