# Supplementary figures and images for: Deciphering pathological behavior of pediatric medullary thyroid cancer from single-cell perspective
Source: PeerJ. 2023 Sep 20;11:e15546. doi: 10.7717/peerj.15546 (PMC10517655; doi:10.7717/peerj.15546)

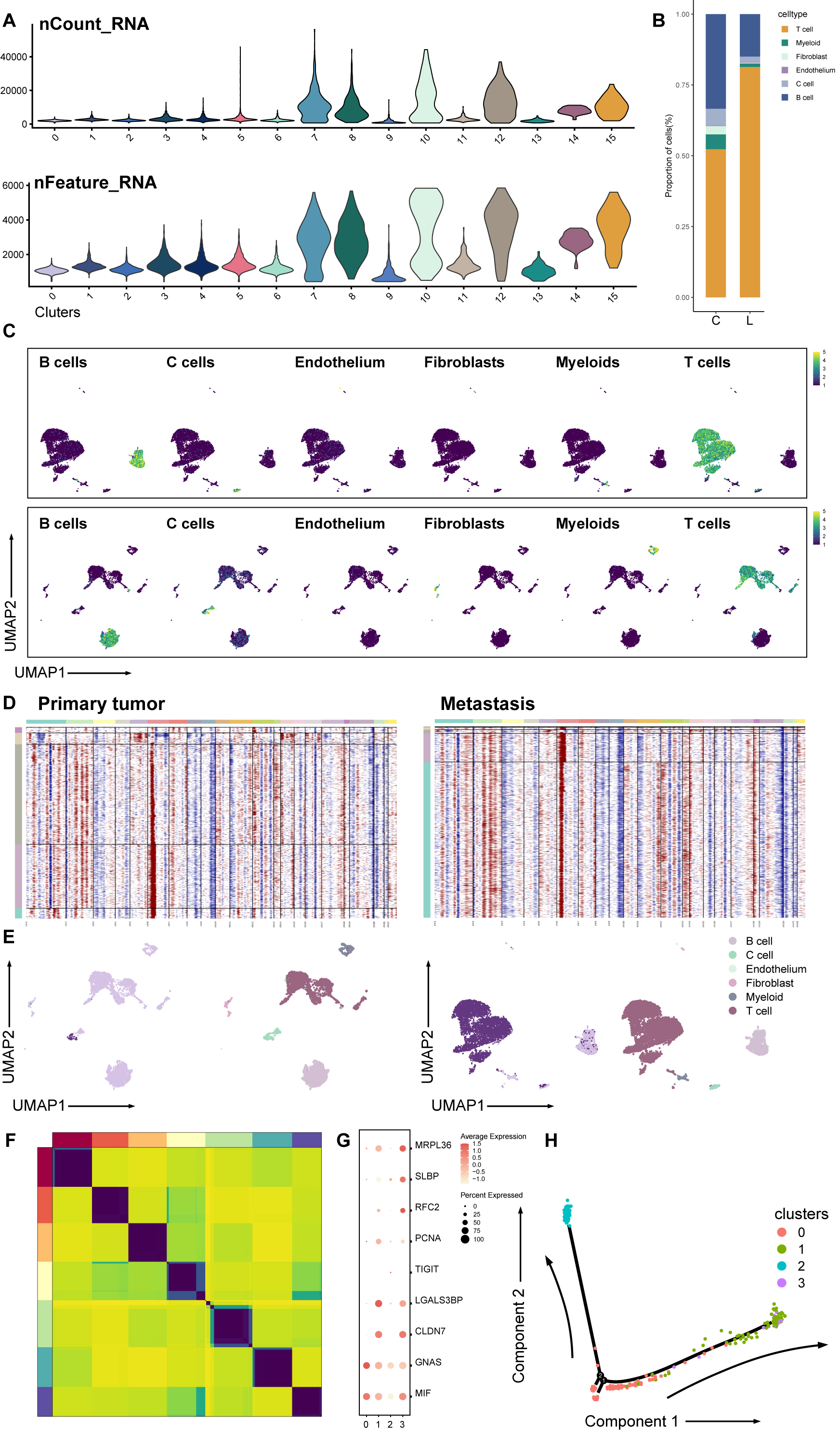

Supplement: Figure S1 — (A) Vlnplots show results of quality control. Upper panel: Vlnplot shows UMI numbers of 3,398 per cell; Downstream panel: Vlnplot shows an average number of 1,418 genes detected in a single cell. (B) Barplot shows proportion of different cell types in primary tumor and metastasis lymph nodes. (C) Upper panel: Umap plots exhibit results of scoring using gene sets of well-known cell type specific markers in primary tumor sample. Downstream panel: Umap plots exhibit results of scoring using gene sets of well-known cell type specific markers in metastasis tumor sample. (D) Left panel: inferCNV result of primary tumor; Right panel: inferCNV result of metastasis lymph tumor. (E) Left panel: Umap plot shows copyKAT prediction result of primary tumor and umap plot shows identification of cell types in primary tumor. Right panel: Umap plot shows copyKAT prediction result of metastasis lymph node and umap plot shows identification of cell types in metastasis lymph node. (F) Heatmap plot shows cNMF results. (G) Dotplot of differential expressed genes in clusters of C cells. (H) Trajectory analysis of C cells of which differentiation directions are indicated by black arrows. [file peerj-11-15546-s001.png]

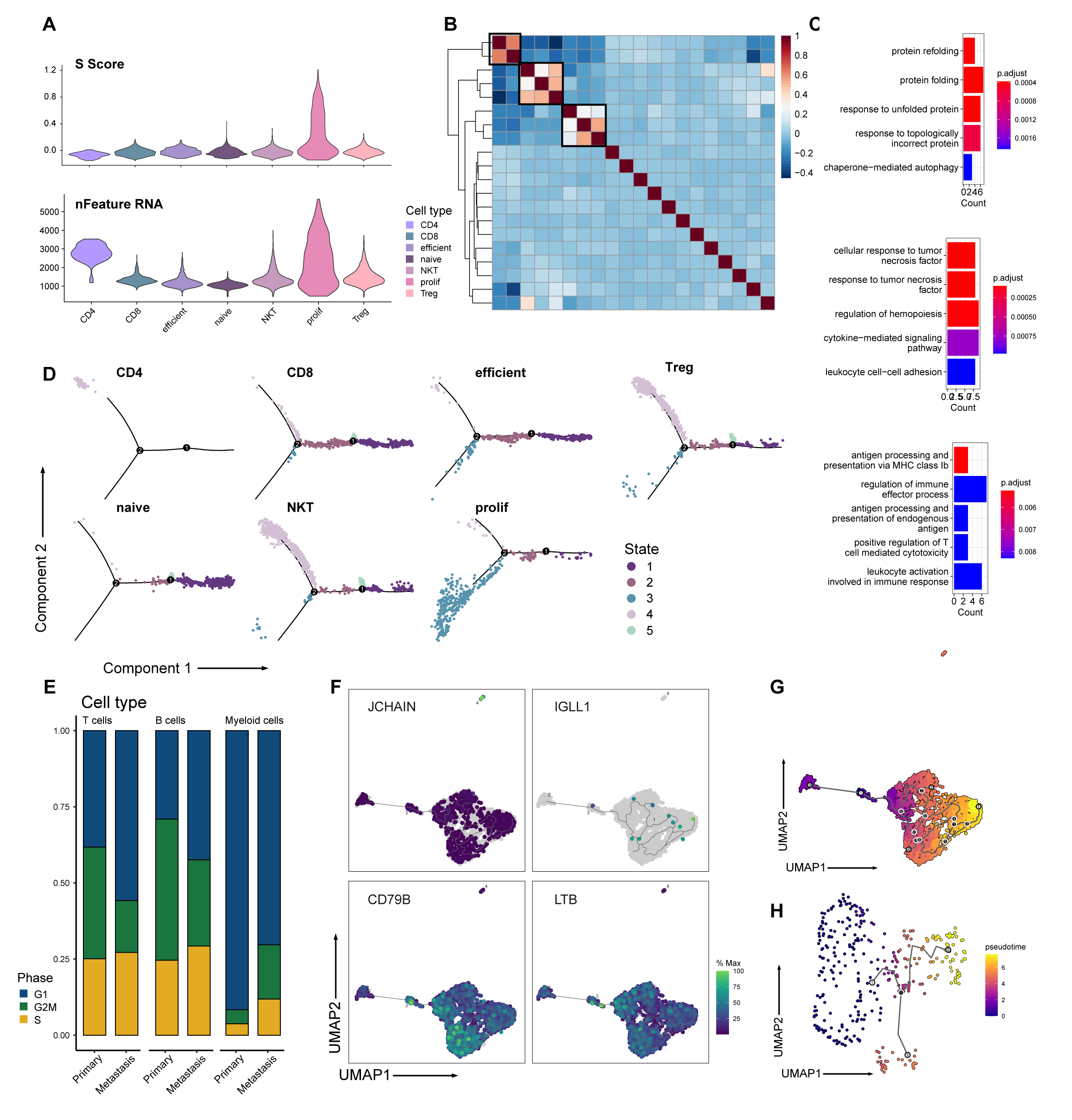

Supplement: Figure S2 — (A) Vlnplots show proliferation state and average gene number of T cell subtypes. (B) Heatmap exhibits that T cells could be divided into 3 meta-programs according to correlation between NMF modules. (C) Barplots show GO result of 3 meta-programs from NMF. Upper panel: module A; middle panel: module B; lower panel: module C. (D) Trajectory analysis results of T cells indicate that subtypes, distinguished by colors, locate in different position of differentiation stages. (E) Barplot exhibits proportion of cells in different phase of cell cycle from primary tumor and metastasis. Proportion of T cells, B cells and myeloid cells are shown separately. (F)Umap plots show expression of JCHAIN, IGLL1, CD79B and CD79A in B cells, showing an obscure tendency in B cell differentiation. (G) Umap shows pseudotime result of B cells. (H) Umap shows pseudotime result of myeloid cells. [file peerj-11-15546-s002.png]

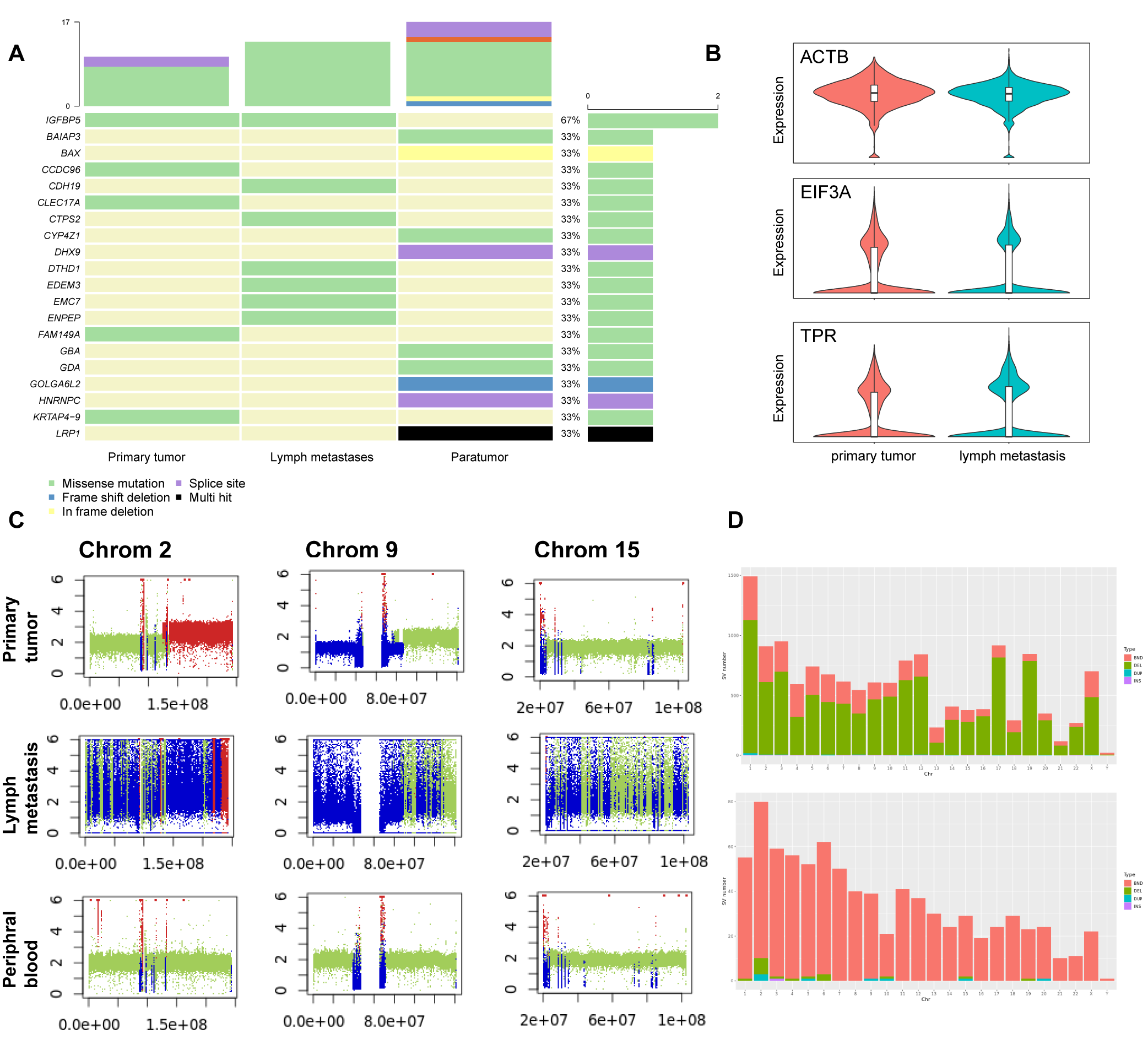

Supplement: Figure S3 — (A) Oncoplot shows mostly mutated 20 genes detected among primary tumor, lymph metastases, and para-tumor samples. (B) Vlnplots of expression of ACTB, EIF3A, and TPR in single-cell data, comparing primary tumor sample with metastasis lymph nodes. (C) Plots of chromosome 2, chromosome 9, and chromosome 15 of the primary tumor sample, metastases lymph nodes, and para-tumor sample show CNV status. (D) Barplots describe SV numbers of each chromosome in metastases lymph node and para tumor samples, and SV types are distinguished by colors. [file peerj-11-15546-s003.png]
